# Stretching Boundaries: Nurses' Perceptions on Job Demands and Resources in Hospital Float Pools

**DOI:** 10.1155/jonm/5468634

**Published:** 2025-09-05

**Authors:** Caroline Fischer, Gréanne Leeftink, Anke Lenferink

**Affiliations:** ^1^Department of Public Administration and Management, University of Kassel, Kassel, Germany; ^2^Interdisciplinary Research Group Technology in Healthcare Transformations, University of Twente, Enschede, the Netherlands; ^3^Department of Industrial Engineering and Business Information Systems, Center for Healthcare Operations Improvement & Research, University of Twente, Enschede, the Netherlands; ^4^Department of Health Technology and Services Research, TechMed Centre, University of Twente, Enschede, the Netherlands; ^5^Department of Pulmonary Medicine, Medisch Spectrum Twente, Enschede, the Netherlands; ^6^Clinical Research Centre, Rijnstate Hospital, Arnhem, the Netherlands

**Keywords:** flexible deployment, float pool, hospital nurse, job demands, job resources, job satisfaction, staffing shortage

## Abstract

**Background:** Float pools are increasingly used in healthcare to enhance staffing flexibility and efficiency. However, the impact of floating on nurses remains underexplored. Challenges may include adjusting to different ward routines and limited team integration.

**Aim:** To explore the perceived demands and resources associated with hospital float pool work, comparing experiences of nurses in intraorganizational pools with expectations of those preparing for interorganizational floating.

**Methods:** This qualitative study, guided by the job demands–resources (JD-R) model, involved semistructured interviews with 27 nurses across five Dutch hospitals. Participants included nurses currently working in intraorganizational float pools and those anticipating working in a float pool across organizations.

**Results:** Nurses in intraorganizational float pools generally reported job satisfaction, experiencing minor demands such as limited team acceptance. Learning opportunities and variation in tasks were key resources. Effective coping was supported by openness, confidence, and communication skills. In contrast, nurses not yet deployed but are anticipating interorganizational floating expected greater demands, including adapting to varying protocols and working across multiple hospital cultures. They emphasized the need for extrinsic resources such as rewards and described personal challenges such as time management and a preference for routine.

**Conclusion:** Interorganizational floating is perceived as more demanding than intraorganizational float pool work. However, experienced nurses often reframe demands as manageable. Findings highlight the importance of a person-centered float pool design that aligns with individual characteristics and support needs. Tailoring float pool policies may enhance job satisfaction, reduce burnout, and support retention among floating nurses.

## 1. Introduction

An aging population with more (chronic) diseases together with shortages of healthcare employees calls for adaptions of working models in healthcare [1, 2]. In response, some healthcare organizations have successfully implemented flexible working models [3], referred to as float pools or flex pools. These float pools can be either implemented within an organization (intraorganizational), so that in-house employees are shared between several units, or across several organizations (interorganizational) [4, 5]. The COVID-19 pandemic highlighted the effectiveness of these flexible working models as hospitals had to shift nurses from less overburdened wards to intensive care units (ICUs) and dedicated COVID-19 wards [6, 7].

In general, float pools are a form of resource pooling, which describes the combination and sharing of organizational resources—such as staff, equipment, or facilities—across units or organizations to improve efficiency, flexibility, and responsiveness to demand [8]. A nursing float pool allows healthcare organizations to make effective and efficient use of the available personnel and infrastructure because it allows flexible responses to fluctuations in care demand and staffing supply [5, 9, 10]. Float pools are often described as a beneficial innovation in nursing management [11, 12].

However, the literature so far disagrees on the microlevel outcomes of float pools. For some nurses, the rotation between hospital departments and/or healthcare organizations might increase job satisfaction because they value the increased autonomy and the opportunity to develop and grow [5, 13–15]. However, frequent rotation can also be demanding as it increases travel time and develops a constant need for adapting to new contexts [16, 17]. These differences might result from the way such float pools are organized, since working arrangements and float pool configurations vary. Weber-Riddener [18], for example, points to the effect of governance of float pools on professionals' engagement.

Based on the job demands-resources (JD-R) model [19–23] working in a float pool may constitute a demand for hospital nurses, based on their individual preferences. Here, demand refers to aspects of a job that drain energy and therefore cause demotivation, dissatisfaction, or eventually burnout. Available resources—aspects that help employees handling these demands—might help employees to handle these job demands, and the organizational design of the float pool [24, 25].

Therefore, in this study, we explored the following questions:

How do hospital nurses experience work in a float pool? Which resources help hospital nurses to cope with the demands of working in the float pool?

To take differences related to the organization of float pools into account and also consider the fact that some of these organizational types are not yet implemented in practice, the following subquestions are posed: *How does the organizational setup of a float pool—such as working within multiple departments of one hospital or across multiple organizations—affect nurses' perceived demands and resources? How does experience affect the nurses' perception of this working arrangement (in contrast to mere expectations)?*

## 2. Background

### 2.1. Resource Pooling and Flexible Deployment of Personnel

Resource pooling is essential in healthcare management to efficiently organize and deliver high-quality care when resources are scarce [8, 26]. It involves sharing or floating of resources within and across healthcare provider systems. Resources within such a pool, e.g., beds, medication, or (cross-trained) staff, are flexibly allocated to the parts of the healthcare system in greatest need to mitigate the effects of variation in care demand and supply [9, 27, 28]. Resource pooling can also help to overcome geographical imbalances in healthcare systems and ensure accessibility of healthcare related to reasonable traveling distances for patients [29, 30].

Float pools can be organized within (i.e., *intraorganizational* pool) or between healthcare organizations (i.e., *interorganizational* pool). It may be organized as a (1) dedicated (internal or external) capacity source, where staff has no home-base unit (i.e., float pool and agency staff), or (2) staff of a dedicated primary home unit that is temporarily deployed to other units in certain circumstances [24, 31, 32]. However, there is no consensus on the best flexible deployment strategy, and combinations of various strategies coexist in healthcare [4]. Moreover, responsibilities of healthcare professionals vary under these flexible deployment strategies. For example, a floating nurse could be fully responsible for a certain patient, providing temporary support only [4], or could be supervised by home-based medical staff [1]. Float pools are also used in residency programs or traineeships for nurses who start working in a hospital [33]. However, introducing interorganizational nurse pools usually requires a period of cross-training before nurses may start working in other hospitals [34]. This includes receiving formal certification but also learning about different equipment used in other hospitals, or processes that differ from what one is used to.

In more general cases, pooling of resources in healthcare improves care delivery [8] as it leads to reduced waiting times, better utilization or occupancy of several types of essential capacities, and a reduction of the need (and therefore the cost) for safety capacity through centralization [9]. More specifically, pooled staffing could also increase the quality of care, as staff has prior experience in various units [5]. In addition, pooling can lead to more balanced workload, patient-to-nurse ratios, and shift lengths [35]. However, due to heterogeneity in care delivery and service targets, such benefits cannot be not guaranteed [31, 37]. Furthermore, (cross-trained) staffing pools potentially increase costs (e.g., due to higher wages), reduce productivity or quality of their work (because they are generalists rather than specialists), and leadership and control gets more complex [9, 28, 38–40].

Flexibly deployed staff have different qualities and skills compared to regular staff, e.g., they need to be cross-trained for several tasks [41] and be able to adapt well to changing work environments [4, 5]. Such needs, however, constitute substantial challenges for nurses. In addition, float nurses report insufficient recognition for their job [42]. There is also a tendency that float nurses are assigned to more difficult patients (e.g., acute patients), must handle more patient movement (admissions, discharges, or transfers) and, generally, provide care to more patients during their shifts than regular nurses [34, 43]. Research so far has provided evidence for nurses' negative feelings regarding float pools which is related to unfamiliarity with the float unit or concerns about competence, ultimately generating stress for nurses [34].

Due to decreasing patient-to-nurse ratios as an effect of pooling, nurses can potentially benefit from more reasonable workload and shift length, along with related beneficial outcomes such as decreased exhaustion and increased job satisfaction [35]. In addition, a higher flexibility in the work schedule is found to be protective of emotional exhaustion, which could be the implication of working in a float pool [36]. Similar research finds that nurses who choose to work in a float pool appreciate the variety of tasks they experience and the exposure to new colleagues, treatments, processes, or medicine that provides them with an opportunity to learning and develop individually [34].

On the other hand, research also shows that pooling can potentially lead to irregular shift patterns which can cause burnout [44]. Such working conditions can lead to recruitment difficulties in flexible deployment [9]. In addition, issues related to collaboration (e.g., lack of sense of community, willingness to be part of such a pool, and IT-solutions incompatible with other systems) or the required specialization (e.g., inadequate professional competence and knowledge regarding practical differences between hospitals and their departments, a lack of standardized way of working) also represent barriers for flexible deployment in healthcare. van Schingen et al. [45] also find that mandatory mobility can be burdensome, especially when it occurs irregular and on too short-term notice. All of these should be managed when creating staff pools [9]. Overall, the literature acknowledges difficulties created by a float pool, especially on the level of nurses involved. There is, however, lack of knowledge on what exactly makes working in a float pool demanding and how the hospitals (e.g., hospital management, supervisor, and float pool managers) could support nurses to cope with these demands.

### 2.2. JD-R Theory

The JD-R model presents a psychological theory that explains both well-being and burnout at the workplace [46–49] and has been proven useful in healthcare to study the effect of organizational support [50], variations in the psychosocial work environment [21, 51], or presenteeism [52] among others. The central assumption is that individual level outcomes have organizational implications as they affect work performance. In the field of healthcare, it was, for example, shown that high demands in the form of work pressure impact quality of care [53], in particular, patient safety [54]. Importantly, Unruh and Nooney [55] and McVicar [56] find that floating shift patterns and shift work in general (next to patient load, work hours, and inadequate orientation) lead to the perception of high job demand and consequently low job control.

The JD-R model builds on earlier frameworks such as the job demand–control–support (JDCS) model [57–60], which emphasized the role of job control and social support in buffering job strain. However, the JD-R model was developed to offer greater flexibility by incorporating a broader range of demands and resources [23, 61], making it especially suitable for diverse and complex work settings such as healthcare and float pooling.

Central to the JD-R model is the idea that although every organization is unique, the working context of all employees can be described in terms of job demands and job resources. While job demands include aspects of the job that drain energy (e.g., role ambiguity, work pressure, and task complexity), job resources refer to aspects that help employees to handle daily job demands (e.g., autonomy, support from supervisor and coworkers, and task significance). This may give the impression that job resources are beneficial for employee well-being while job demands are not. However, job demands are not necessarily harmful. They only become negative when meeting them requires too great an effort, from which employees cannot recover [19]. Thus, job demands can be both challenging and hindering (e.g., [62, 63]). According to van den Broeck et al. [64], job demands appeal to employees' curiosity, competence, and thoroughness, thereby eliciting approach-oriented coping styles, which in turn are contributing to achieving work goals. Hindering job demands, in contrast, imply adverse consequences for employees. Cavanaugh et al. [65] (p. 68) define them as “work circumstances that involve excessive or undesirable constraints that interfere with or inhibit an individual's ability to achieve valued goals.”

Based on this ongoing theoretical discussion, this study explores whether flexible deployment constitutes a challenging or hindering demand for hospital nurses, including how this perception is created. The experience of working in a float pool—in contrast to mere expectations of such an experience—might correct false (positive or negative) assumptions.

**Figure 1:**
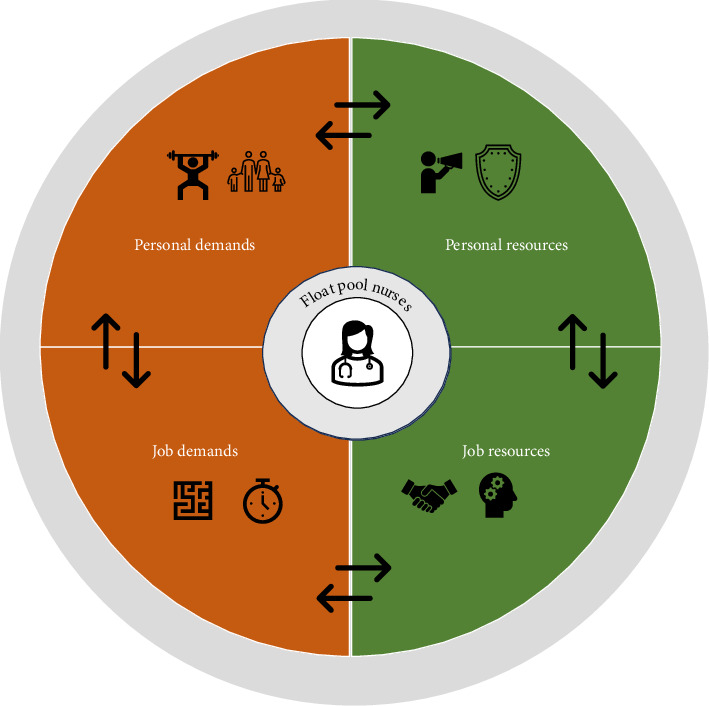
Interaction of personal and job demands and resources.

An essential building block of JD-R theory is the interaction of job demands and resources (see Figure 1). On the one hand, job resources can help buffer negative results of job demands, such as strain [66] preventing further outcomes such as turnover [67–69]. For example, Zito et al. [70] find that job resources help in creating a flow at work, which decreases exhaustion and the effect of other job demands on exhaustion in nurses. Ghislieri et al. [71] show that organizational support can help to buffer job demands that would otherwise lead to work family conflict. On the other hand, challenging job demands can strengthen positive effects of job resources on employee well-being [66]. This means that resources shape the work context of individuals and determine effects of job demands. For example, complex tasks can result in positive and negative work-related outcomes. It is the combination of task complexity with valuable feedback and autonomy (job resources) that can lead to higher levels of energy and increase the likelihood of reaching difficult goals. Based on these considerations, this study investigates which job resources help hospital nurses to deal with the demand of working in a float pool.

JD-R theory has been expanded to also include *personal* demands and *personal* resources (next to *job* demands and *job* resources) [20]. Personal demands have been defined as “the requirements that individuals set for their own performance and behavior that force them to invest effort in their work and are therefore associated with physical and psychological costs” [72] (p. 751). Typical examples of personal demands are workaholism and personal traits such as emotional instability and perfectionism. In contrast, personal resources refer to positive self-beliefs that are linked to resilience and individuals' sense of their ability to control and impact their environment successfully, e.g., self-efficacy, optimism, and self-esteem (Hobfoll et al., 2003). Working in a float pool not only affects work life but also private life, due to commuting and scheduling of shifts. Therefore, this study will also consider personal demands adding to the work demand of flexible deployment with personal resources acting as a potential buffer. Furthermore, personal resources supporting or hindering nurses to work in a float pool are explored.

Altogether and based on the described knowledge gaps, this study aims to clarify the specific job and personal demands that arise among nurses through working in a hospital float pool as well as to analyze resources to buffer these demands. This will provide better understanding of how organizational structure influence nursing practice, staff well-being, and workforce sustainability. The findings will also give insights into how management can better support flexible nursing roles through organizational design, leadership approaches, and targeted resource allocation.

## 3. Methods

### 3.1. Design and Sampling

This study employs a qualitative study design with the aim to gain in-depth understanding of how nurses experience job demands and resources within the specific organizational context of hospital float pools. The JD-R model guided the design of our data collection and the deductive part of the subsequent analysis, which was accompanied by an inductive analysis as a second step.

A multiple-case study design was chosen because this is particularly well-suited for exploring complex, context-dependent phenomena in real-life settings [73–75], and it allows for comparisons across diverse hospital settings, enabling the identification of both shared and context-specific patterns. The chosen hospitals vary in size (1000–3500 employees, 200–600 hospital beds, and 50,000–400,000 outpatients) and complexity of delivered care (general hospital and top clinical hospital) and are located in different regions of the Netherlands. By embedding two samples within several hospitals, the design further allows exploration at both the organizational and individual level, which is appropriate given the study's interest in both systemic arrangements (e.g., type of float pool) and subjective experiences.

We interviewed nurses from five Dutch hospitals from May to June 2022 (two samples [76, 77]). One hospital is represented in both samples, but different nurses from this organization were included in Samples 1 and 2. Nurses were eligible if they were either working in an intraorganizational float pool, or working in an organization that is considering to install an interorganizational float pool, and employed by one of the included hospitals. Nurses not employed by the hospital were excluded.

In total, 10 float nurses from three hospitals were interviewed in Sample 1 (respondents 1–10 F), with the aim to elicit nurses' *experienced* demands and resources regarding flexible deployment in an *intraorganizational* float pool. These nurses already work in an intraorganizational float pool (hence, they are flexible within the hospital and not bound to a specific ward, but they are not supposed to work in other hospitals). All float nurses were invited to participate via their respective float pool coordinators. Nine interviews were conducted online and one on-site in the hospital.

In Sample 2, 17 nurses from three hospitals were interviewed (respondents 11-27 NF). These hospitals are geographically close to each other and plan to cooperate in an interorganizational float pool, where nurses work in different hospitals within this float pool. Since the envisioned float pool is planned for ICU nurses only, these specific nurses are interviewed in this sample. The aim was to identify those nurses' *expected* demands and resources regarding flexible deployment in an *interorganizational* float pool. In Sample 2, nurses were invited to participate via their department heads. All interviews were conducted at the family rooms of the wards.

### 3.2. Data Collection

Individual semistructured interviews were conducted using a Dutch-language interview guide (the English translation is found in Appendix A). Interviews covered the following themes: personal background information, flexible deployment, demands, resources, and benefits of and for float pools. For example, demands were explored by using questions, such as “What are the elements in your work that you experience as annoying that are related to the float pool?” Among others, resources were explored by asking open-ended questions such as “What are the benefits of working in a float pool?” Additional probing questions were used as needed to elicit richer responses.

Interviews were conducted either online (via video conferencing) or in person, depending on participant preference and availability. They lasted on average 30–45 min. All interviews were audio-recorded and transcribed verbatim using automatic language recognition software (Amberscript). Verbatim transcription was used to ensure accurate capture of participants' language, phrasing, and emotional tone—critical for preserving meaning and enabling nuanced thematic analysis [78, 79]. Consistency across interviews was ensured by using the same interview guide and by having the same researcher conduct all interviews in each of the samples.

### 3.3. Data Analysis

Interview data were analyzed according to a six-stage thematic analysis [78]: (1) immersion, (2) generating initial codes, (3) searching for and identifying themes, (4) reviewing themes, (5) defining and naming themes, and (6) drafting the report. In the first three stages, a mix of deductive and inductive coding was used to label text fragments of the interview transcriptions. Deductive coding was guided by our theoretical basis pointing to demands and resources, and inductive coding happened as a next step within these categories. Subsequently, these codes were compared and translated into overarching themes guided by theory. This process was done for three interviews by two researchers independently and disagreements were discussed. For the final coding schemes of both samples, see Tables 1 and 2. Data analysis was supported by ATLAS.ti software.

### 3.4. Ethical Considerations

The study was approved by the ethical committee of the University of Twente (BMS ethics committee, Domain Humanities and Social Sciences) at 22.3.2022 (protocol nr. 220,100) and 14.4.2022 (protocol nr. 220,191). The Medical Research Involving Human Subjects Act did not apply to the study. Written informed consent was obtained from all participants prior to the interviews.

## 4. Findings

In this section, after generally describing the interviewees, the experienced demands and resources when working in an intraorganizational float pool are discussed, followed by the expected demands and resources when working in an interorganizational float pool.

### 4.1. Characteristics of Interviewees

In Sample 1, all nurses are directly employed in a float pool; some of them also work to varying extends in other float pools, as sports trainers or in a self-employed status in parallel. Two of these 10 nurses work full time, and 8 part-time (i.e., less than 36 h per week). However, all of them also report that working time differs a lot throughout the year; sometimes they work full-time and in other times less. In Sample 1, it was not possible to collect age-data, since this would deanonymize the nurses in the float pools. However, tenure within the float pool is on average 4 years, with nurses working just a year and other working already for 21 years in the pool.

In Sample 2, 12 (out of 17) interviewed nurses work part-time within their hospitals (i.e., less than 36 h per week). Five nurses had a self-employment status next to their part-time contracts. These nurses can apply for additional shifts in other healthcare organizations and have, therefore, experience in adjusting their schedules but do not work in a float pool. Nurses in Sample 2 have a mean age of 41 years (range from 26 to 56 years) and work for their current employer on average for 17 years (range from 4 to 40 years).

### 4.2. Sample 1—Demands and Resources Experienced in an Intraorganizational Pool

#### 4.2.1. Experienced Demands

Table 1 displays various demands that nurses experience when working in a float pool. In Sample 1, six job demands (lack of communication, lack of acceptance in the team, differences in protocols and procedures, differences in medical terms, inability to maintain skills, and differences in guidance and leadership) have been identified. Notably, only job demands have been mentioned by the participants, but no personal demands. Nurses mentioned that most of these demands appeared in the beginning of working in a float pool, since they had to get used to the hospital and the workload. This is especially true for demands that were clustered under the category of *flexibility and adaption*. Over time, these hindering and stress-inducing demands seem to fade away or turn into challenging demands, at least according to their individual perception. *“So, I believe the workload is high in the beginning. Looking at it now, after a year, it's much lower. As you gain experience, you know what to do, how to handle things, and the sequence.”* (Respondent 1 F). However, demands related to a poor organization of floating and loneliness do not turn into challenges but stay hindrances which need to be actively reduced by the respective hospitals. All groups of demands mentioned by the float nurses are described in more detail in the following.

A *lack of communication* was shown to be the most common demand. One respondent mentioned that sometimes the department where a float pool nurse was scheduled was not aware that the nurse would be coming. Such a lack of communication and coordination results in wasted time and costs creating frustration among pool nurses. Moreover, it also creates frustration among other nurses.

Another reoccurring theme is related to the *acceptance of the float nurse in the team*. Float pool nurses switch departments regularly and therefore also must work with different teams at every department. The data show that this can cause the float pool nurses a feeling of being an outsider. Nurses mention that the other nurses are very welcoming most of the time. However, float nurses still experience some difficulties since they frequently must ask questions if they do not know something and can feel like they ask too much. The other employees are busy and already familiar with the systems and are not always amenable to helping pool nurses.

*Differing levels of knowledge and skills* was also mentioned as a demand by respondents. Hospital departments have their own organizational structures and procedures, and the float pool nurses must quickly orient themselves in this structure. This can be time-consuming and challenging especially when a nurse is switching between different medical fields. From this situation, pool nurses experience feelings of uselessness. However, this demand could be easily buffered by an introduction to routines and essential knowledge, as mentioned by the participants.

In every department, a certain professional language is used in the team which might be unfamiliar to a float nurse. This is especially the case for *medical terms* and related abbreviations. Acquiring relevant terminology can take time and needs persistence but can also appear as being ignorant. Moreover, this job demand can be a risk for patients, for example, a misunderstood note can lead to inappropriate treatment or medication and jeopardizing quality of patient care.

A related concern was to *maintain (medical) skills*. Due to the ongoing rotation, float pool nurses do not work regularly or consistently in one medical field, performing specific tasks more seldom than regular nurses do. They fear the related deskilling. This can lead to a decline in confidence in their own skills and knowledge. One respondent mentions the following: *“Sometimes I do not work two or 3 weeks and then I feel like I lose a bit of skill”* (Respondent 5 F). However, this is a fear of many professionals when they become generalists, as float nurses usually do.

Last, another demand mentioned is the *difference in clinical guidance and formal supervision* in the individual departments. By switching departments, nurses must continuously adapt to (new) leadership styles and interaction in teams. This also includes guidance by peer nurses. Such differences can lead to frustration because one is unaware whom to approach for a specific problem, or nurses might struggle to accommodate differing leadership styles.

#### 4.2.2. Experienced Resources

Nurses working in a float pool also perceive diverse benefits that serve as resources to cope with the demanding situation. In Sample 1, three job resources (learning opportunities, schedule flexibility, appreciation, and valuation) have been identified as well as three personal resources (openness, confidence, and communication skills) that might buffer the negative effects of flexible deployment in float pools. These resources are displayed in Table 1.

The *opportunity to learn* is the most frequently mentioned resource. Pool nurses become knowledgeable about specific departments but also the entire hospital. Those nurses consider this as valuable for their further career and a basis for promotion. Another related benefit mentioned is learning new skills and developing into generalists.

Furthermore, the *flexibility* of having autonomy over their schedule is appealing for nurses who have other responsibilities outside of the hospital. Although personal demands (e.g., balancing private and working life) were not mentioned explicitly as a demand, the reference to this resource could mean that it successfully buffers the negative pressures of, for example, care responsibilities.

Appreciation of float pool nurses is also mentioned as a resource by the respondents. It is important for the float pool nurses to feel appreciated by the general medical departments as well as the float pool team. Some respondents mentioned that being valued by the departments they work in motivates them a lot and helps them to cope with drawbacks of pool working.

In addition, personal resources have been mentioned, one of which was the *capability to quickly adapt* to a new situation. If a nurse adapts easy to new situations, they can cope with the negative implications of flexibility and adaption and turn this demand into a challenge thus reinforcing the benefits of flexible deployment. It was also mentioned that a float pool nurse needs to be (1) *confident* in their own skills and knowledge, (2) transparent about their own knowledge, and (3) willing to ask questions. Consequently, the respondents mentioned that effective *communication skills* serve as a personal resource for pooling.

### 4.3. Sample 2—Expected Demands and Resources in an Interorganizational Float Pool

#### 4.3.1. Expected Demands

The demands that nurses expect to experience when working in a float pool in the future differ from the demands reported by nurses currently employed as float pool workers (see Table 2). The main difference is that in Sample 2 both job and personal demands are mentioned. Four job demands (differences in protocols and procedures, lack of acceptance in the team, differences in complexity of healthcare, and uncertain times) have been identified as well as two personal demands (time investment, need for routines, and stability) that might create negative effects of flexible deployment in float pools.

Both groups of nurses (Samples 1 and 2) agree on the (potential) demand of *differences in protocols and procedures* that may lead to confusion, extra learning time, and potential errors. The participants mention that familiarity with materials, machines, and procedures is especially important in intensive care where timely and appropriate actions are needed.

Like Sample 1, respondents in this group also mention that *belonging to a team* could be hard for a float pool nurse. This is especially true for nurses from smaller hospitals, when floating in large hospitals which could be impersonal and lonely. Furthermore, respondents also mentioned that such an alienation impacts the felt responsibility for the team and the degree of motivation to engage in organizational citizenship, e.g., to take up additional tasks. “*Now, there are a lot of colleagues who have attention fields [specific responsibility] for various topics.* For example*, one is working in a dialysis working group, the other is committed to infection prevention. We do this in addition to [our] regular work. If I start working half of my contract in other hospitals, then at some point I might not feel responsible for that attention field anymore.*” (Respondent 18 NF).

The *difference in levels of complexity* of healthcare in different hospitals was another job demand additionally brought up in this sample. This demand is related to *interorganizational float pools* and pointed to differences between, for example, university hospitals offering more complex care and dealing with more innovative tools and procedures, in comparison to regional hospitals. Respondents find it important to work in an environment with a high complexity of care and experience that as a challenge. If they are needed in a hospital with less complex care and/or patients, they fear they will be underwhelmed and have fewer learning opportunities. Hence, for nurses working in a complex environment, it is less attractive to switch for longer periods into less complex environments because they fear losing their skills and knowledge. In contrast, nurses from general hospitals mentioned that working with a higher level of complexity would motivate them to temporally work in another hospital to learn. In addition, younger nurses find higher complexities of care more attractive than older nurses.

The nurses also mentioned a desire to work in a known work environment. This appears to stem from the time during the COVID-19 pandemic when pool nurses were required to work on short-term notice in unfamiliar hospitals. They stated that they still felt overworked and burntout from the pandemic. Those who were still recovering from this busy period said that they would not be very enthusiastic to work in other hospitals. Working in an interorganizational float pool does not seem attractive in uncertain times.

In terms of personal demands, the time investment and personal needs for routines and stability are mentioned by the interviewed ICU nurses. The first demand is related to the private situations of the nurses, for example, they are less flexible and have less *time to commute* because of care tasks at home. Nurses working near their home are not motivated to travel further than to the current employer. In addition, nurses with children or a higher age want to travel less.

The personal need or tendency toward ability and routines appears to be related to age and work experience. Older nurses indicate that they are stressed by familiarizing themselves with new equipment, systems, or processes and are therefore less motivated to join an *interorganizational float pool*. Similarly, most of the interviewed nurses mentioned seeing their older colleagues struggle with innovations in their work unit. In addition, physical limitations that come with greater age are mentioned as being a hindrance to nurses to work in varying medical settings.

#### 4.3.2. Expected Resources

ICU nurses mentioned similar resources to those reported by experienced float pool nurses, particularly when asked about factors that could facilitate working in a float pool regardless of the previously mentioned demands (Table 2). They name four job resources (learning opportunities, schedule flexibility, rewards, and acceptance in the team) and two personal resources (openness and confidence).

*Learning and experience* as a benefit and motivating factor from working in different hospitals is also mentioned as a resource. Most nurses find a training period in advance important for familiarizing with the new context being flexibly deployed. The interviewed nurses agree that it is important and interesting to them to exchange knowledge with colleagues from other hospitals. Although most nurses are enthusiastic about learning, some do not want to follow obligatory training to obtain additional formal qualifications. This is expected to be too burdensome.

Similarly, nurses without experience with working in a float pool agree that *schedule flexibility* can also be considered as a benefit leading to the ability to better cope with the demands stemming from such a working model. However, it is also pointed to the need to have a stable home-base. *“I would prefer to stay working at my own hospital while working some single shifts in other hospitals. If you are working somewhere else frequently or for a longer period, I think you really miss a lot of what is going on in your own team. I think that connection with your own team is very important”* (Respondent 12 NF).

In addition, the nurses expect tangible *rewards and compensation* to be job resources related to working in a float pool. Incentives as a resource have been mentioned in all interviews, pointing to an extrinsic motivation of the participating nurses. Especially nurses with an additional self-employment status mention that their wages are higher than those of a contracted nurse. For them, it is not attractive to cut back self-employment to join a float pool. *“If they would compensate us for working flexible at* [hospital 1 to 3]*, I would be more open for it. I understand that it will not be in proportion to what I earn as a freelancer. I think a financial compensation would motivate people, because they will be rewarded for the extra effort they give.”* (Respondent 21 NF). Others mention higher commuting allowances or time-for-time arrangements, additional days off or rather intangible incentives such as better working conditions or appreciation from colleagues.

The latter aspects point to another theme that emerged from the data: *acceptance in a team*, which was also mentioned by experienced float pool nurses (there as a demand because of the lack of acceptance) to be a key factor in float pools. Participants state that they have high trust in their current teams and strong bonds with the doctors in their departments. They also indicate that these relationships would be needed in teams in which they float to be able to work better. Participants mention that it would help them to feel safer and more welcome as a floating nurse.

Concerning personal resources, participants in Sample 2 agree with Sample 1 that *adaptability* and *confidence* in own skills are important individual prerequisites for successfully working in a float pool. Nurses without experience of working in a float pool in contrast do not specifically point to communication skills.

**Figure 2:**
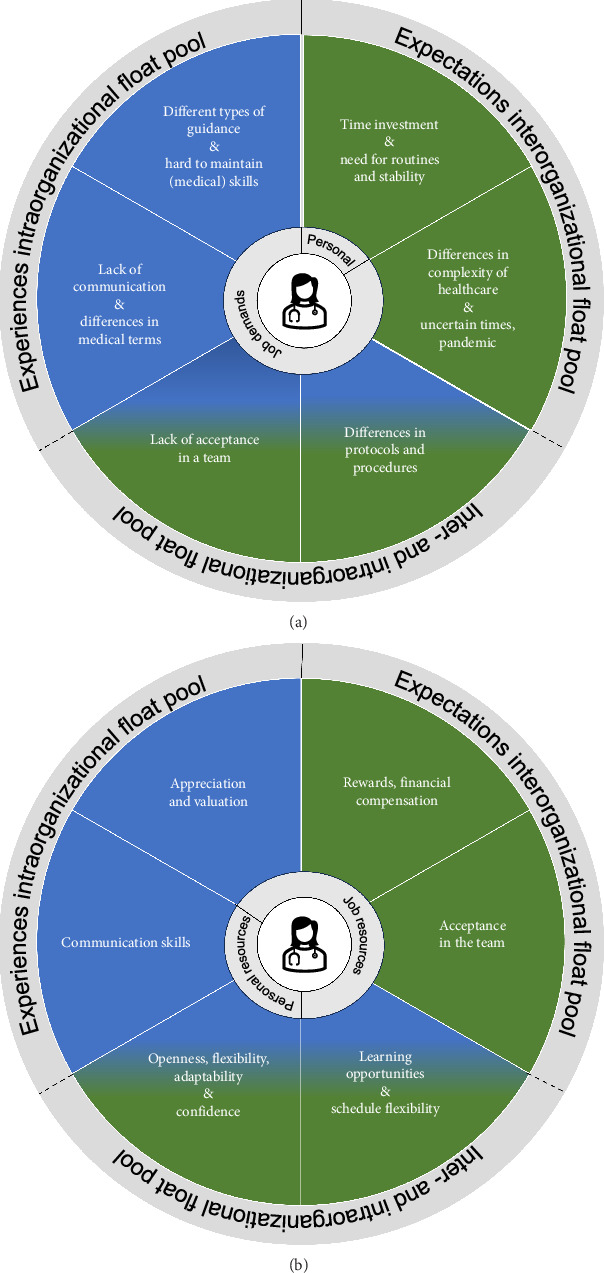
(a) Personal and job demands identified from the intra- and interorganization float pool. (b) Personal and job resources identified from the intra- and interorganization float pool.

Figure 2 summarizes all obtained results on the identified personal and job demands (Figure 2(a)) and personal and job resources (Figure 2(b)).

## 5. Discussion

The interviews revealed that most nurses already working in an *intraorganizational float pool* are satisfied with their job. They see only minor demands of flexible working models, such as differences in medical terms and materials and a lack of coordination of the float pools in general. Since they do not belong to a specific team, float pool nurses sometimes feel like an outsider, leading to work alienation and frustration. However, nurses not already working in a float pool and interviewed concerning their expectations about an *interorganizational float pool* also hold negative opinions about being flexibly deployed. They indicate demands such as inconsistency in training and certificates across the different hospitals. In addition, they highlight the need for incentives, such as a pay raise, to cope with the demanded flexibility, pointing to an extrinsic motivation for such a role. This might become problematic in the long term because professionals without sufficient intrinsic motivation, purely motivated for a job extrinsically, are more likely to leave a job when first turbulences occur, to underperform, or at least not to engage in organizational citizenship behavior [80, 81].

Both the nurses already working in float pools and those who were asked about their expectations of working in these float pools identify similar (potential) resources that buffer demands raised by working in a float pool. Hence, both groups of nurses are well informed about advantages and sources of help to cope with work stress. However, they evaluate the relationship between demands and resources differently. That might be related to the additional demand than an interorganizational float pool adds. Interorganizational pooling increases traveling time and might also make the demands mentioned by nurses working in an intraorganizational float pool more pronounced because processes, terms, structures, and leadership styles might differ even more between hospitals than just between different wards. Studies on different medical disciplines also show that there is indeed a high heterogeneity between different hospitals in a country, which can be related to different quality standards, available machines, materials, procedures, expertise, or routines in applied care (for the Netherlands, for example, [82–84]). Skipper [85] developed a framework of competences as a basis for assigning nurses to different wards and guiding float pool nurses' education to deal with challenges related to differing knowledge and routines.

However, this difference can also be explained by other intervening variables, such as the nursing profession, the interviewers, or the influence of experience. The latter one is particularly notable in the result that the experienced float pool nurses did not report personal demand, but rather resources that helped them to cope with personal demands. Also, over time, float pool nurses might get used to initially hindering demands which either turn into a challenge then or do not appear anymore at all. Hence, the finding by Larson et al. [34] concerning negative feelings of float pool nurses stemming from concerns about missing competence and unfamiliarity and the related work stress should disappear over time.

This temporal dimension adds a novel idea to the differentiation into hindering and challenging demands in the JD-R literature [86, 87]—namely, a potential development of hindering into challenging demands over time (implying that a development in the opposite direction might also occur). The temporal dimension calls for further research following respondents over time. This study suggests that such a switch from hindrance to challenge occurs via learning, socialization within an unfamiliar environment, and an increasing identity with the float pool, pointing to the important role of organizational and group identity [88]. Further quantitative studies should test this mediating relationship.

Another perspective is that nurses inexperienced with float pools might report expectations driven from prejudices, bad word of mouth, and negativity bias [89, 90], and experience corrects these false negative assumptions. This corresponds with the so-called expectation disconfirmation model [91, 92], which suggests that individuals form expectations prior to an experience, and their satisfaction is influenced by whether the actual experience confirms or disconfirms those expectations. The model differentiates empirical expectations, which form through experience and normative expectations based on values and assumptions. If nurses have the chance to collect empirical expectations, their perspective on float pooling might be more nuanced and realistic. Sometimes one must be thrown in at the deep end to enjoy swimming. Hence, future research should test whether our negative results for *inter*organizational float pools occurred due to missing experience of the interviewed nurses and how experience of working in an *inter*organizational float pool impacts the outcomes.

Note that the decrease in demands over time reflects a subjective perception of demand. This could be due to, e.g., increased coping mechanisms after working in a float pool. However, what might also be noticed here—when seeing the differences between experienced and nonexperienced float pool nurses—is a so-called survivorship bias [93]. The interviewed nurses working in a float pool might just be the ones who “survived” working there and did not already switch jobs after a shorter period of time (or did not even apply for such a position). Hence, that they perceive less demands and more benefits from working in a float pool could be the result of a self-selection mechanism (especially based on personal demands related to working in such a model) due to the voluntary participation in such a pool. This could point to a particular personality that creates a better person-job fit in a float pool. Identifying the individual characteristics that may lead to better outcomes and increased job satisfaction, in line with person-job fit theory, is an open area for further research. However, from this study, one can draw implications for the recruitment in a float pool, namely, that professionals should at least bring the personal resources found in this study to cope with the demands of a float pool.

Coming back to the difference between *intra-* and *inter*organizational float pools and the finding that the latter seems more challenging for the nurses working within them is a learning point for the further development of healthcare systems. This study started with the assumption that float pools are increasingly needed in the future to deal with increasing care demands in parallel to less available staff and the consequence for more efficiency. *Intra*organizational float pools can primarily be used to fill in open spots due to illnesses or similar reasons for short-term absence [4]. However, they are usually more useful to substitute general activities instead of expert positions because it is even less efficient to have experts purely on standby in case someone gets sick. *Inter*organizational float pools in contrast can deliver exactly this highly specialized expertise by, for example, sharing a specific expert or a procedure between several hospitals [30, 94]. In terms of labor shortage, these experts will get even more rare in future, and sharing is therefore a reasonable approach. If one wants to, therefore, build a float pool where specific expertise is shared, one must take the demands and needed resources found within this study into account, to make such an endeavor successful. Also, the created efficiency in terms of labor costs is questionable. As seen from the results of this study, float pool professionals might need to receive extra pay or other incentives to be willing to work in such a working environment. However, if not money but staff shortages are the core problem to solve, and a float pool is using the available short personnel in a better distributed way.

*Inter*organizational float pools are expected to get more prominent, and more healthcare systems will move toward planning and delivering care in networks on a regional level, integrating, e.g., community care, home care, and general practitioners with care in hospitals [94, 95]. In future, float pool nurses might not only float between healthcare providers of the same type but also between different healthcare providers. This will add another level of complexity in relation to needed skills and competencies but potentially also motivating these float nurses by enabling a higher continuity of care for specific patients.

To decrease demands arising from commuting longer distances between healthcare providers, these *inter*organizational float pools can also be supported or in some cases even substituted by virtual care [96]. Consultations with patients, monitoring their vital signs or just providing an expert opinion are tasks that can easily be provided in a virtual way, in contrast to the manual labor involved in healthcare. In that regard, as many hospitals and other care providers are implementing virtual care in virtual care units or digital service centers, it might make sense to combine such an innovative structure with the implementation of a float pool. Future research needs to investigate the dynamics of such a combination.

Our study offers at least three important contributions to the literature. *First*, this study adds a qualitative underpinning to the JD-R model that is until date mostly tested in a quantitative way. This qualitative approach helps us to shed some more light on characteristics of float pools that makes working in them either satisfying or more demanding. *Second*, by introducing different perceptions of experienced and expected demands, the study adds another theoretical layer to the JD-R model, taking learning from experience into account. The results showed that expectation management needs to take place when introducing a float pool because nurses mostly share burdensome expectations that eventually turn out to be way less demanding when experienced in practice. *Third*, this study takes an interdisciplinary approach to the analysis of float pools. It traditionally analyzes a topic discussed in the operations management and planning literature with an organizational–psychological lens. This combination enables a holistic understanding of microlevel implications of flexible deployment in healthcare.

Practical implications for hospital and float pool managers follow from these theoretical contributions. The analysis of resources and demands leads to a list of beneficial characteristics of float pools that should be fostered, such as learning opportunities and schedule flexibility, and hindering characteristics that should be reduced, such as communication problems or belongingness to the team. If the design of float pools recognizes these findings, it happens in a person-centric way—also taking the needs of the healthcare professionals into account instead of only patients' needs. If working conditions for float pool nurses can be made better in this way, patients also benefit from a safer environment.

### 5.1. Limitations

Although this study adds important insight into the difference between organizing float pools within one or between multiple organizations and on the temporal development of the perception of demands, it also comes with limitations. *First*, this work shows the combination of two independent studies in interorganizational and intraorganizational settings. The studies were combined, as comparing similarities and differences in the two samples was promising. However, the study was not able to complement the sample with a third study with nurses with experience working in an interorganizational float pool. Future research must prove this by analyzing such a rare type of interorganizational float pool (potentially in another national context). Future research should follow the implementation of an interorganizational float pool on a regional level closely.

Second, the two different samples have been interviewed with slightly different questions and by different interviewers, who might have influenced the participants' answering behavior. However, this variety of questions was also needed, as the nurses worked in vastly different situations (float pool vs. not float pool and general nurse vs. ICU nurse), and the aim was also to capture differences in the design of float pools (interorganizational vs. intraorganizational). In addition, the selection of interviewees did not take place randomly but was dependent on the supervisors of the respective units in the hospitals, who might have recruited participants in a biased way. However, since the primary aim of qualitative research is not to reach generalizable conclusions but rather to generate novel insights, this might be negligible.

### 5.2. Conclusions

Nursing float pools are frequently suggested as the panacea to deal with staff shortages and varying demands in healthcare systems. Research often simulates and models efficiency gains but oversees the burdens that such a working model produces for the personnel. Although it is known that float pool nurses experience stress, it remained unclear so far, which job and personal demands led to this work strain. This study found that interorganizational floating is more demanding than being flexibly deployed within one organization. However, experience helps to turn hindering demands into challenging ones or diminish demands at all.

This study's findings can help identify essential employment resources to best support professionals employed in float pools, such as hospital nurses. This, in turn, can support further tailoring the implementation of float pools within and between organizations.

Practitioners can learn from the results whom to recruit for which type of float pool, namely, individuals with personal resources such as communication skills and less personal demands such as a lack of adaptability. At the same time, this research highlights that float pool nurses have to be appreciated by their colleagues and incentivized for the additional effort to make working in such an arrangement sustainable.

From a theoretical perspective, this study provides a qualitative underpinning to the JD-R model which so far has only been tested in a quantitative way. In this study, the introduction of different perceptions of experienced and expected demands adds another theoretical layer to the model. The study also adds to the literature on flexible work by studying a systematic form of flexible employment related to work time, work place, and work tasks, as well as the literature on the ‘dark sides' of work flexibility—the potential negative consequences [97, 98] by adding nuance to the aspects of flexible work that raise demands for flexible professionals.

## Figures and Tables

**Table 1:** Demands and resources experienced in an intraorganizational float pool.

Category	Code	Definition	Exemplary quote
*Demands*			
Job demands	Lack of communication	Insufficient communication between home units, float pool managers, and/or float nurses	“I find the ad hoc responses a bit challenging; you receive a message for the same day, and that requires quick adjustments. And if there's something in the flex, I don't really have a supervisor, and I find that a bit of an issue. I would really like to have a supervisor who listens to our wishes, but now you often get feedback like, “you should talk to the department's supervisor”” (respondent 6 F).
	Lack of acceptance in the team	Feeling of not belonging to the team, difficulties with team members	“In a float pool you never quite belong. You're not really part of the team. While they are very involved and sweet, and it's not like they do not see you, but you are not one of the team” (respondent 5 F).
	Differences in protocols and procedures	Differences in organizational structure and processes, storage and handling of material and tools, implicit knowledge about routines	“You have all new colleagues, new syndromes and everything is in a different place” (respondent 3 F)
	Differences in medical terms	Differences in abbreviations, professional language used in the department, problems in task-related communication	“In some departments you really have to google everything the doctors write down” (respondent 2 F).
	Hard to maintain (medical) skills	Feeling of incompetence and/or declining confidence in own knowledge and skills in specific medical fields, perception of lack of routine	“I think you miss some depth sometimes. When you're permanently assigned to a department, you focus on specific medical conditions within that whole department, and you can really delve into them and learn a lot. In the flex pool, it's less so because you're on each department for a shorter time. In 2 months, you can't learn as much as someone who's permanently on the department” (respondent 2 F).
	Differences in clinical guidance and formal supervision	Differences in responsibilities and leadership roles, different leadership styles	“Then she gets an explanation from another colleague who is just different or does it differently or they get to another department where they do things differently, so that is sometimes difficult for starting nurses” (respondent 10 F).

*Resources*			
Job resources	Learning opportunities	Practical experience and learning by exposure to new medical fields, new wards, new ways of working, personal growth in terms of collaboration and other soft skills	“When you're hired, they offer you the opportunity to work in various departments. This is incredibly beneficial for you as you get to see the entire hospital” (respondent 1 F).
	Schedule flexibility	Enjoying more flexibility regarding work time due to the float pool	“Well, back then, it wasn't a choice because I couldn't do night shifts during the week anymore since I became a parent in the meantime. So, they said, “sorry, then you have to go on call because then you can say no”” (respondent 6 F).
	Appreciation and valuation	Nurses feel appreciated by their leader, their team, their organization, their patients for working in a float pool	They are always very happy that you are willing to work. Well, you get the same appreciation from the organization as anyone else. When it comes to bonuses during the pandemic or a gift or a card, we get those too (respondent 5 F).

Personal resources	Openness, flexibility, adaptability	Nurses describe themselves as flexible, able to adapt to new situations, resilient to stress, or think that is needed or beneficial for the float pool	“You definitely need to be flexible. I hear that a lot from people; that you can handle that, that you enjoy doing it. Many people say: I can't imagine switching every day; you really need to be able to handle that” (respondent 2 F).
	Confidence	Nurses are self-confident, believe in themselves and their abilities, or think that confidence in own skills and knowledge is needed or beneficial for the float pool	“It's also about how you deal with it yourself. I can handle stress quite well, so if I end up in a busy department, I think, “okay, let's get through the day.” I don't let it affect me too much” (respondent 3 F).
	Communication skills	Nurses are good communicators, don't hesitate to offer or ask for help, easy to get in contact with others, or think that is needed or beneficial for the float pool	“The most important thing is that you should be a proactive person. If you don't dare to take the initiative, it won't be an ideal place for you” (respondent 4 F).

**Table 2:** Demands and resources expected by float pool nurses in interorganizational float pools.

Category	Code	Definition	Exemplary quote
*Demands*			
Job demands	Differences in protocols and procedures	Differences in organizational structure and processes, storage and handling of material and tools, implicit knowledge about routines	“If this float pool will eventually be created, I think you also must align protocols and procedures, or they should at least try to make them resemble. I think there is still quite a discrepancy in procedures. For example, I know [Hospital 1, 2, and 3], have very different ways of looking at ventilated patients” (respondent 19 NF, hospitals anonymized)
	Lack of acceptance in the team	Feeling of not belonging to the team, difficulties with team members	“The ICU at [Hospital 1] is divided. So, in principle these are small departments, but […] the team there is huge. That makes it more impersonal. I know a story of an ex-colleague of mine, who went to [Hospital 1]. […] One time he went away for 4 months. When he came back, no one asked him about it. At one point somebody asked him if he went on vacation because they did not see each other for several weeks. He found that very annoying at the time” (respondent 16 NF, hospitals anonymized).
	Differences in complexity of healthcare	Differences in level of care delivered by hospitals, related feeling of higher and lower-level tasks, fear to unlearn or to get downgraded	“What I like about a large ICU, for example this one in [City 1], is that you have a lot of diversity and that you get complex patient cases. For example, someone was brought in by helicopter yesterday who has all kinds of problems and in which I can take a directing role as a nurse. You don't have that possibility in [City 2 or 3] because the helicopter does not bring the trauma patients there” (respondent 19 NF, names of cities anonymized).
	Uncertain times, pandemic	Concerns about new working environments in uncertain times, like pandemics, wish to get some stability when work demands increase	“During the first COVID-19 wave, we had some very ill patients. You were responsible for four ICU patients instead of two. In a situation like that, nothing is better than to be able to work in a familiar environment where you immediately know where to find everything and where you know exactly what to do and how the equipment works” (respondent 20 NF).

Personal demands	Time investment	Critical standpoint about additional time needed to work in a float pool, especially related to commuting time	“Now that I have children, I must arrange the babysitter. I must take travel time into account. […] The kids must be brought somewhere else very early in the morning because I must go to work. […] So that also contributes to the fact that I would like to work somewhere closer to home” (respondent 14 NF).
	Need for routines and stability	Personal need to work in a stable environment, to keep routines, to be stressed by change	“I have many colleagues who have always worked in this hospital. Some might not have any work experience in other hospitals. They would not be open to something like this” (respondent 22 NF).

*Resources*			
Job resources	Learning opportunities	Practical experience and learning by exposure to new medical fields, new wards, new ways of working, personal growth in terms of collaboration, and other soft skills	“I have always said that we can learn a lot from working at different hospitals. People often complain about their work, but by working at several places you will learn that it is not better over there” (respondent 15 NF).
	Schedule flexibility	Enjoying more flexibility regarding work time due to the float pool	“It would be more attractive to me if I would not have to work night shifts at other hospitals, because I am so done with the night shifts. So, if you are for example allowed to plan your own shifts and fill in the gaps and that these hours would count within my contract. That would be attractive to me because I can fill in my shifts more flexibly” (respondent 22 NF).
	Rewards, financial compensation	Incentives to motivate nurses working in a float pool, such as pay raises, commuting allowances, or intangible benefits	“I think if there would be anything more to it, to working flexible in other hospitals, which would have a positive influence. Something in the form of money or rewards. I think that would make working elsewhere within your field of work a little more attractive” (respondent 24 NF).
	Acceptance in the team	Feeling of belonging to the team, getting along with team members	“I really enjoy working in a team and also getting to know the people you work with a little better. (…) And you see that it is quite close here, that people know each other very well. I think that certainly improves cooperation. That you look after each other” (respondent 17 NF)

Personal resources	Openness, flexibility, adaptability	Nurses describe themselves as flexible, able to adapt to new situations, resilient to stress, or think this is needed or beneficial	“Yes, I would really like working in a hospital with a higher complexity of healthcare. You can only learn more from it. So yes, I would see that as something positive.” (respondent 14 NF)
	Confidence	Nurses are self-confident, believe in themselves and their abilities, or think that is needed or beneficial	“With my experience, I think, well, I should, be able to do well working at another department or hospital. So, in that sense it is not a barrier for me and I am curious about it” (respondent 14 NF)

## Data Availability

The data are not publicly available due to privacy restrictions. However, the article tries to show as much direct data as possible to make the analysis as transparent as possible.

## Appendix A

**Interview guide Sample 1**

1. In which hospital do you work?
2. Do you work part-time or full-time in the float pool?
3. How long have you been working in the float pool?
4. In which departments do you work?
5. Why did you choose to work in a float pool?
6. How do you experience the work pressure as a float pool nurse? Why high/low work pressure?
7. Can you describe a general week as a float pool nurse?
8. Do you need more/other skills as a float pool nurse? If yes, which skills?
9. What are the main differences between float pool nurses and regular nurses?
10. How many times do you switch departments in general?
11. Do you have input in your schedule? (Preferences)
12. What are the elements in your work that you experience as annoying that are related to the float pool?
13. Which obstacles do you experience that influence your work in the float pool?
14. What are the benefits of working in a float pool?
15. What are important aspects that a float pool must consist of?
16. Are float pool nurses rewarded enough for the work that they are doing in your opinion?
17. Which rewards would stimulate nurses to participate in a float pool?

**Interview guide Sample 2**

1. In which hospital do you work?
2. Do you have a full-time or part-time contract with the hospital?
3. How many years have you been working in this specific hospital?
4. How many years have you been working as a nurse? And how many years on the ICU?
5. Is this the only organization or institution you currently work for?
6. Imagine a situation in which the xxx, yyy, and zzz (anonymized) would decide to create a float pool of ICU nurses, whereby you have the possibility to work on all three locations. You will receive additional training based on your work experience, so that you get familiar with all three locations and will be able to fully participate in the three teams. What do you think about this plan?
7. What are the factors that influence your perspective with regard to the situation I just described?
8. To what extent does the perspective of your colleagues regarding flexible deployment at xxx, yyy, and zzz (anonymized) influence your own perspective?
9. What is your perspective regarding flexible deployment if many of your direct colleagues would be open for flexible deployment?
10. What is your perspective regarding flexible deployment if few of your direct colleagues would be open for flexible deployment?
11. Imagine the following two situations. The first situation is that you will be deployed at one of the three hospitals for half a year. After this period, you will return to your own hospital. The second situation is that you will, for example, work in your own hospital for around 20 h and in one of the other two hospitals for 16 h. Do you prefer one of these two situations? And why do you prefer this situation?
12. What would be your point of view regarding flexible deployment when a new epidemic or pandemic would arise?
13. To what extent does the size of the hospital or ICU ward you work for influences your point of view regarding flexible deployment at xxx, yyy, and zzz (anonymized)?
14. How is the complexity of healthcare in the hospitals you do not have a contract with influence your perspective on flexible deployment at xxx, yyy, and zzz (anonymized)?
15. To what extent does the culture within the hospital or ICU ward you work at influences your point of view regarding flexible deployment on a different location? The culture can be described as, for example, the ‘we-feeling,' shared norms and values, the way employees are treated.
16. To what extent does the phase in life you are in influence your perspective on flexible deployment? For the phase in life, you can think about, for example, how much work experience you have, if you have children or if you are a caregiver to someone.
17. To what extent is having more variety in work an influential factor for your perspective on flexible deployment at xxx, yyy, and zzz (anonymized)? More variety in work could be, for example, more variety in tasks, working with different colleagues or working in a different environment.
18. To what extent do stress and work pressure influence your perspective on flexible deployment at xxx, yyy, and zzz (anonymized)?
19. How does the amount of traveling time to xxx, yyy, and zzz (anonymized) influence your perspective on flexible deployment at these three locations?
20. If you would receive compensation for becoming flexibly deployed, would that change your perspective on flexible deployment at xxx, yyy, and zzz (anonymized)? You could think about compensation for additional travel expenditures or extra income, for example.
21. Are there any other factors that influence your perspective that have not been mentioned? If yes, what are these factors?
